# Intramitochondrial Ascorbic Acid Enhances the Formation of Mitochondrial Superoxide Induced by Peroxynitrite via a Ca^2+^-Independent Mechanism

**DOI:** 10.3390/ijms18081686

**Published:** 2017-08-02

**Authors:** Andrea Guidarelli, Liana Cerioni, Mara Fiorani, Orazio Cantoni

**Affiliations:** Dipartimento di Scienze Biomolecolari Università degli Studi di Urbino “Carlo Bo”, 61029 Urbino, Italy; andrea.guidarelli@uniurb.it (A.G.); liana.cerioni@uniurb.it (L.C.); mara.fiorani@uniurb.it (M.F.)

**Keywords:** peroxynitrite, ascorbic acid, mitochondrial superoxide, DNA damage, mitochondrial dysfunction, complex III

## Abstract

Exposure of U937 cells to peroxynitrite promotes mitochondrial superoxide formation via a mechanism dependent on both inhibition of complex III and increased mitochondrial Ca^2+^ accumulation. Otherwise inactive concentrations of the oxidant produced the same maximal effects in the presence of either complex III inhibitors or agents mobilizing Ca^2+^ from the ryanodine receptor and enforcing its mitochondrial accumulation. l-Ascorbic acid (AA) produced similar enhancing effects in terms of superoxide formation, DNA strand scission and cytotoxicity. However, AA failed to enhance the intra-mitochondrial concentration of Ca^2+^ and the effects observed in cells supplemented with peroxinitrite, while insensitive to manipulations preventing the mobilization of Ca^2+^, or the mitochondrial accumulation of the cation, were also detected in human monocytes and macrophages, which do not express the ryanodine receptor. In all these cell types, mitochondrial permeability transition-dependent toxicity was detected in cells exposed to AA/peroxynitrite and, based on the above criteria, these responses also appeared Ca^2+^-independent. The enhancing effects of AA are therefore similar to those mediated by bona fide complex III inhibitors, although the vitamin failed to directly inhibit complex III, and in fact enhanced its sensitivity to the inhibitory effects of peroxynitrite.

## 1. Introduction

Peroxynitrite, the coupling product of nitric oxide and superoxide, promotes extensive damage on diverse biological molecules including lipids, proteins and DNA [1,2,3]. While these effects can be directly mediated by peroxynitrite, it appears clear that the oxidant also promotes secondary damage by triggering events resulting in the time-dependent formation of different damaging species. As an example, most of the DNA single-strand breakage generated by peroxynitrite is in fact mediated by superoxide-derived H_2_O_2_ [4,5]. Superoxide formation ensues in a reaction in which ubisemiquinone serves as an electron donor and is followed by its prompt dismutation to H_2_O_2_, which can now exit the mitochondria, reach the nucleus and promote site-specific hydroxyl radical-mediated DNA cleavage [4]. Importantly, formation of superoxide requires electron transport in the mitochondrial respiratory chain and is critically regulated by the availability of mitochondrial Ca^2+^ [5]. In particular, our studies performed in U937 cells showed that peroxynitrite mobilizes Ca^2+^ from the ryanodine (Ry) receptor (RyR) and that the cation subsequently accumulates in the mitochondria, thereby enhancing the rate of superoxide/H_2_O_2_ formation. This response was therefore named CRDM (Ca^2+^-/respiratory chain-dependent mechanism) [6]. Consequently, cells become resistant to CRDM upon supplementation with rotenone (an inhibitor of complex I) or myxothiazol (an inhibitor of the electron flow from the reduced coenzyme Q to cytochrome c_1_ preventing the formation of ubisemiquinone), or when made respiration deficient via prolonged growth in the presence of ethidium bromide [4]. Alternatively, a resistance phenotype was acquired via inhibition of Ca^2+^ mobilization from the RyR [5], or through down-regulation of Ry receptors, as it occurs during differentiation of promonocytic U937 cells to monocytes [7]. Likewise, human monocytes or macrophages, while not expressing the RyR, displayed collateral resistance to CRDM [6,7].

An interesting development of the above studies was that either of the two components of the above mechanism may, under specific conditions, acquire independency from the other one. These two mechanisms shared with CRDM the characteristic of being saturable, but required remarkably lower concentrations of peroxynitrite [6].

In particular, a mechanism uniquely dependent on Ca^2+^, named Ca^2+^-dependent mechanism (CDM), was induced by otherwise inactive concentrations of peroxynitrite in cells supplemented with agents mobilizing Ca^2+^ from the RyR, as caffeine (Cf), resulting in enforced mitochondrial Ca^2+^ accumulation. CDM, while insensitive to inhibition of electron transport, was sensitive to Ry. As a corollary, respiration-deficient cells were sensitive to CDM, unlike human monocytes and macrophages that were resistant to this mechanism.

A respiration-dependent mechanism (RDM) was instead observed in cells supplemented with the complex III inhibitor antimycin A [6,8] and low concentrations of peroxynitrite, with hardly any effect on various parameters, including Ca^2+^ homeostasis [8]. RDM was therefore sensitive to rotenone, myxothiazol and the respiration-deficient phenotype but insensitive to high concentrations of Ry. Consistently, the cocktail antimycin A/peroxynitrite produced also effects through RDM in human monocytes and macrophages [6].

We recently reported that l-ascorbic acid (AA), a very well established antioxidant [9,10], produces unexpected effects in cells exposed to otherwise inactive concentrations of peroxynitrite [11,12]. Supplementation of the vitamin was indeed associated with enhanced mitochondrial superoxide formation in response to otherwise inactive concentrations of peroxynitrite [11]. These effects were not associated with the autoxidation of the vitamin and were in fact mediated by a specific mechanism provided of the following characteristics: (1) it was saturable and maximally induced by as low as 3 µM AA [11]; (2) it was entirely attributable to the mitochondrial fraction of the vitamin [11]; (3) it was sensitive to rotenone, myxothiazol or the respiration-deficient phenotype [11].

We also provided an explanation for the observed requirement of very low concentrations of the vitamin. The cell type employed in these studies expresses high levels of sodium-AA co-transporter 2 (SVCT2), which, with a Km value of 8.4 ± 0.76 μM, allows effective cellular uptake of the vitamin [13]. This event is then associated with an uptake of the vitamin in mitochondria, once again mediated by SVCT2, which surprisingly works with a Km comparable to that of the plasma membrane, despite the restrictive conditions in terms of Na^+^ or Ca^2+^ intracellular concentrations [13]. It follows that, even at low extracellular levels of AA, the vitamin is continuously taken up by the cells and further accumulated in their mitochondria.

The present study was performed with the aim of investigating the mechanism involved in the enhancing effects of AA. We found that the vitamin enhances peroxynitrite-dependent superoxide/H_2_O_2_ formation via a Ca^2+^-independent mechanism, remarkably similar with that triggered by bona fide complex III inhibitors, therefore classifying as RDM. The effects of the vitamin, however, were not associated with inhibition of oxygen consumption, as in the case of the complex III inhibitors; rather, intramitochondrial AA, while failing to affect oxygen consumption, enhanced the sensitivity of complex III to the inhibitory effects of peroxynitrite.

## 2. Results

### 2.1. Relationships between Mitochondrial Superoxide Formation, with the Ensuing Downstream DNA Strand Scission, and Mitochondrial Ca^2+^ Accumulation in Cells Exposed to AA and Peroxynitrite

The initial experiments were performed with the aim of investigating the Ca^2+^ requirements of the effects mediated by a low concentration of AA and peroxynitrite with the use of a comparative approach. More specifically, critical findings documenting the Ca^2+^-dependence of CRDM and CDM, as well as the independence of RDM, were compared with the mechanism under investigation.

**Figure 1:**
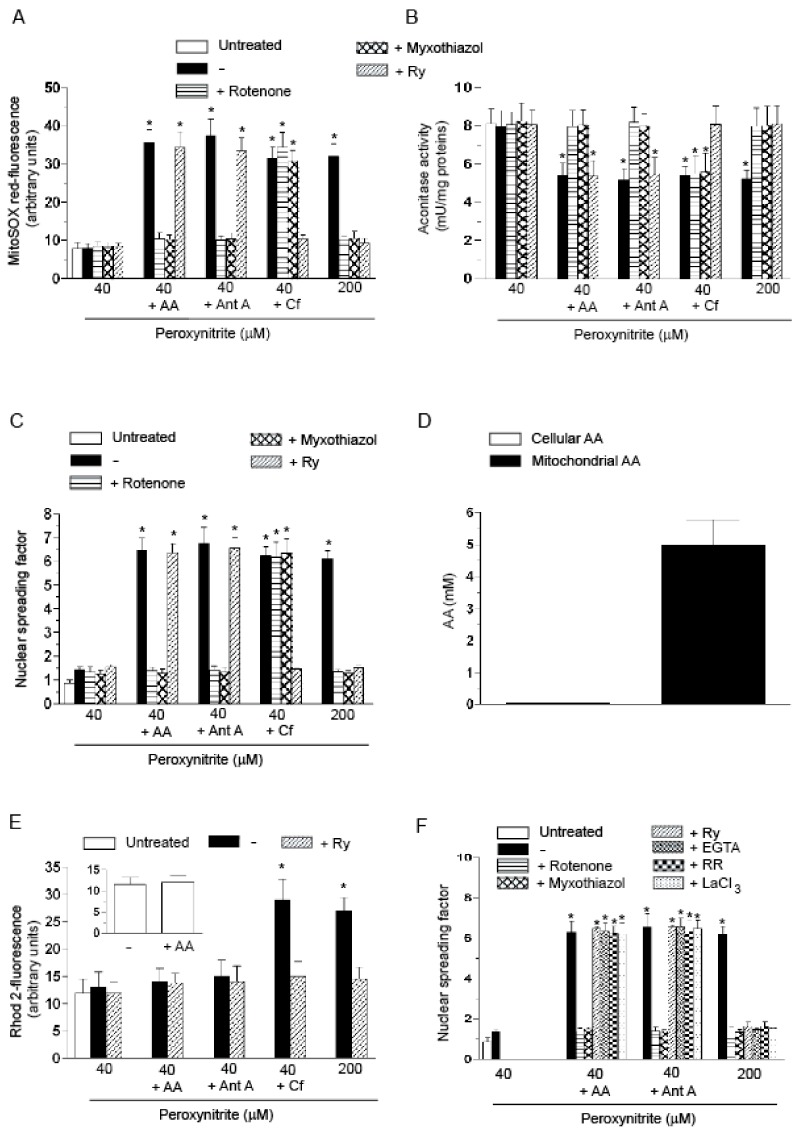
The enhancing effects of AA in U937 cells exposed to low concentrations of peroxynitrite: evidence for a Ca^2+^-independent mechanism. (**A**–**C**) The cells were treated as shown in the figure and then exposed for: 10 min (**A**,**B**); or 30 min (**C**) to the indicated concentrations of peroxynitrite. AA (3 µM) was given to the cells 15 min prior to peroxynitrite. Pre-exposure to antimycin A (Ant A, 1 µM), or Cf (10 mM), was instead of only 5 min. In some experiments, rotenone (0.5 µM), myxothiazol (5 µM) or Ry (ryanodine) (20 µM) were added to the cells 5 min prior addition of AA, Ant A or Cf. After treatments, the cells were analyzed for: MitoSOX red-fluorescence (**A**); aconitase activity (**B**); and DNA strand scission (**C**); (**D**) the cells were exposed for 15 min to AA and immediately analyzed for their cellular and mitochondrial content of the vitamin; (**E**) Rhod 2-AM (acetoxymethyl) pre-loaded cells were treated for 10 min, as indicated in the figure, and analyzed as detailed in the Materials and Methods Section. The inset shows the Rhod 2-fluorescence response observed in cells exposed for 15 min to 0 or AA; (**F**) cells were permeabilized with digitonin and exposed for 10 min to the indicated concentrations of peroxynitrite alone or associated with rotenone, myxothiazol, Ry, EGTA (ethylene glycol-bis(β-aminoethylether)-*N*,*N*,*N*′,*N*′-tetraacetic acid) (10 µM), RR (ruthenium red) (200 nM) or LaCl_3_ (100 µM). In some experiments, the cells were exposed to AA prior to permeabilization. In other experiments, the cells were supplemented with Ant A immediately after permeabilization. Results represent the means ± SD calculated from at least three separate experiments. * *p* < 0.001 as compared to untreated cells (one-way ANOVA followed by Dunnett’s test).

A 10 min exposure to 40 µM peroxynitrite fails to promote MitoSOX red-fluorescence (Figure 1A), or inhibition of aconitase activity (Figure 1B), in cultured U937 cells. MitoSOX red is a fluorescent probe detecting superoxide in the mitochondria of live cells [14] and aconitase, mostly localized in mitochondria [15], is an enzyme particularly sensitive to inhibition by mitochondrial superoxide [16,17]. Hence, under these conditions, peroxynitrite fails to promote mitochondrial superoxide formation, instead detected using greater concentrations (e.g., 200 µM, Figure 1A,B) of the oxidant. Consistently, there was no evidence of DNA strand scission after a 30 min exposure to 40 µM peroxynitrite (Figure 1C). Under the same conditions, treatment with 200 µM peroxynitrite instead caused DNA damage.

These observations were followed by the demonstration that the above events are instead observed in cells pre-loaded for 15 min with as low as 3 µM AA and then treated with 40 µM peroxynitrite. Identical enhancing effects were obtained under conditions in which AA was replaced with either 1 µM antimycin A (RDM) or 10 mM Cf (CDM). As previously determined [4,6], the above effects were all sensitive to rotenone and myxothiazol, with the exception of those mediated by Cf/peroxynitrite. Ry abolished the effects mediated by Cf/peroxynitrite, or by the high concentration of peroxynitrite alone, with hardly any effect detected on the paradigms involving exposure to the oxidant associated with either AA or antimycin A.

In these results, we can find a first indication of the Ca^2+^ independence of the enhancing effects of AA, based on the sensitivity to inhibition of electron transport and insensitivity to Ry. These responses were identical to those obtained with RDM, which is also Ca^2+^ independent.

The low concentration of AA employed in the above experiments was selected on the bases of previous studies showing that, under these conditions, the vitamin promotes maximal enhancing effects in cells exposed to peroxynitrite [11,12]. In addition, as shown in Figure 1D, this short-term exposure to AA promotes a low intracellular accumulation associated to a remarkably greater concentration of the vitamin in the mitochondrial compartment, as a result of the high expression of functional high affinity SVCT2 in these organelles [13,18]. Notably, however, this condition produced hardly any effect on the mitochondrial Ca^2+^ concentration, as assessed with the fluorescent probe Rhod-2 acetoxymethyl ester (AM) (inset to Figure 1E). The same lack of effects was observed in cells exposed to 40 µM peroxynitrite with or without AA, or antimycin A (Figure 1E). A different outcome was instead provided by experiments in which the cells were exposed to Cf/peroxynitrite, or to a high concentration of the oxidant (200 µM). The Rhod-2-derived fluorescence response associated with either of these two latter conditions was suppressed by Ry.

These results are in keeping with those presented above and put more weight on the hypothesis of the Ca^2+^ independence of the enhancing effects of AA. An additional indication in this direction is provided by experiments in digitonin-permeabilized U937 cells. The results illustrated in Figure 1F indicate that 40 µM peroxynitrite fails to promote DNA cleavage also under these conditions. AA supplementation prior to permeabilization was however associated with a remarkable DNA strand scission, sensitive to rotenone or myxothiazol and unaffected by Ry. Importantly, DNA damage was insensitive to 10 µM ethylene glycol-bis(β-aminoethylether)-*N*,*N*,*N*′,*N*′-tetraacetic acid (EGTA), a calcium chelator, to 200 nM ruthenium red (RR), that under these conditions specifically prevents mitochondrial Ca^2+^ uptake [19], as well as to lanthanium ions (100 µM), known to competitively inhibit mitochondrial Ca^2+^ uptake [20]. Interestingly, these treatments produced an identical response under conditions in which the DNA strand scission was obtained by replacing AA with antimycin A. As a final note, each of the above treatments prevented the DNA strand scission induced by 200 µM peroxynitrite.

The experiments reported in this section collectively provide a strong indication that a low concentration of AA enhances the effects of peroxynitrite via a Ca^2+^-independent mechanism, as we previously demonstrated for RDM.

### 2.2. Mitochondrial Superoxide Formation and DNA Strand Scission in Human Monocytes and Macrophages Exposed to AA and Peroxynitrite

**Figure 2:**
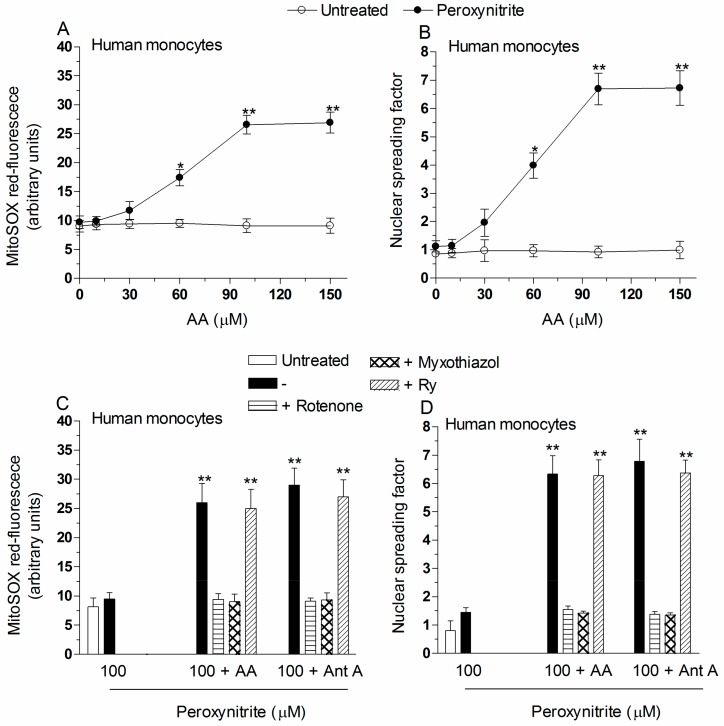

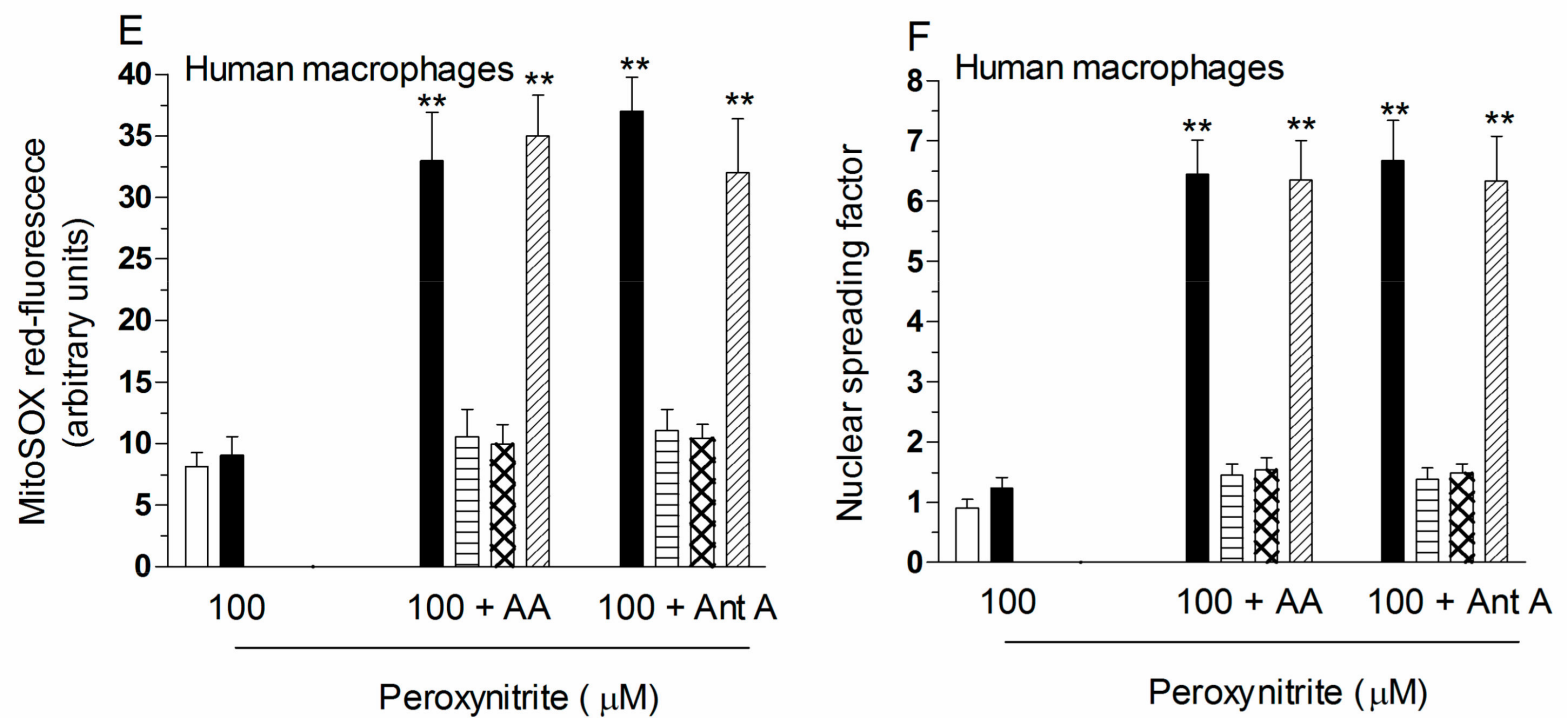
The enhancing effects of AA are also observed in human monocytes or macrophages exposed to peroxynitrite. Human monocytes were pre-exposed for 15 min to increasing concentrations of AA and then treated for a further: 10 min (**A**); or 30 min (**B**) with 100 µM peroxynitrite. After treatments, the cells were analyzed for: MitoSOX red-fluorescence (**A**); and DNA damage (**B**). Results represent the means ± SD calculated from at least three separate experiments using monocytes from three different donors. * *p* < 0.01 or ** *p* < 0.001 as compared to untreated cells (two-way ANOVA followed by Bonferroni’s test). Human monocytes (**C**,**D**); or macrophages (**E**,**F**) were pre-exposed for 15 min to 100 µM AA, or for 5 min to antimycin A (Ant A), and then treated for a further: 10 min (**C**,**E**); or 30 min (**D**,**F**) with 100 µM peroxynitrite. In some experiments, rotenone, myxothiazol, or Ry, were given to the cultures prior to peroxynitrite. After treatments, the cells were analyzed for: MitoSOX red-fluorescence (**C**,**E**); and DNA damage (**D**,**F**). Results represent the means ± SD calculated from at least three separate experiments using monocytes (or monocyte-derived macrophages) from three different donors. ** *p* < 0.001 as compared to untreated cells (one-way ANOVA followed by Dunnett’s test).

In our previous studies, we found that intrinsically effective concentrations of peroxynitrite mobilize Ca^2+^ from the RyR of U937 cells and that the cation is subsequently cleared by the mitochondria to then promote events resulting in superoxide formation [5]. It follows that cells which do not express the RyR, such as the U937 cells differentiated in culture, as well as human monocytes and macrophages, acquire resistance to peroxynitrite [7]. Human monocytes were indeed resistant to the MitoSOX red fluorescence (10 min, Figure 2A) and DNA damaging (30 min, Figure 2B) responses induced by 100 µM peroxynitrite. Under the same conditions, however, monocytes acquired sensitivity when supplemented with increasing concentrations of AA. Note that greater concentrations of the vitamin were required in comparison with U937 cells to produce significant effects in monocytes. In particular, pre-exposure to 3 µM AA, while mediating maximal enhancing effects in U937 cells (Figure 1A–C and Reference [11]), failed to produce detectable responses in monocytes (Figure 2A,B). To detect the same enhancing effects obtained in promonocytic cells, it was necessary to pre-load monocytes with 100 µM AA. In addition, under the same conditions of vitamin pre-exposure, the results obtained with human macrophages were in line with those from experiments using monocytes (Figure 2E,F). Most importantly, however, the enhancing effects mediated by AA on superoxide formation (Figure 2C,E) and DNA damage (Figure 2D,F) were in both cell types prevented by rotenone, or myxothiazol, and unaffected by Ry. These results are therefore once again in line with those obtained with antimycin A/peroxynitrite. An additional important consideration is that, in the absence of peroxynitrite, even high concentrations of AA failed to enhance in the MitoSOX red-fluorescence response, or induce DNA strand scission, since dithiothreitol (DTT) was added during vitamin C pre-loading to prevent its autoxidation.

In summary, as previously observed with RDM [4,8], supplementation of AA circumvents the resistance of monocytes and macrophages to the effects of peroxynitrite and triggers events likely mediated by Ca^2+^-independent mechanisms.

### 2.3. AA Promotes Peroxynitite-Dependent Mitochondrial Permeability Transition and Cytotoxicity via Ca^2+^-Independent Mechanisms

**Figure 3:**
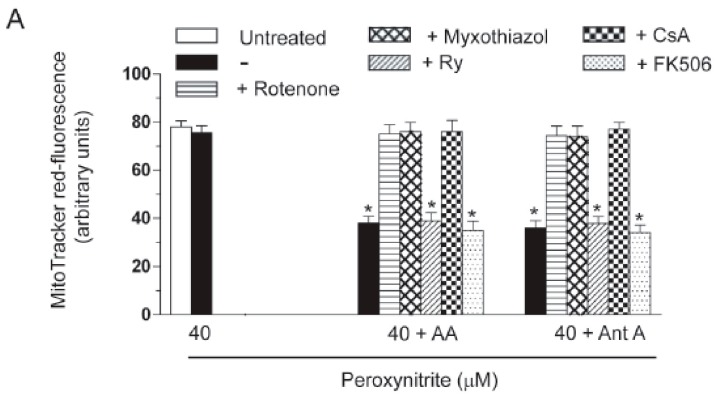

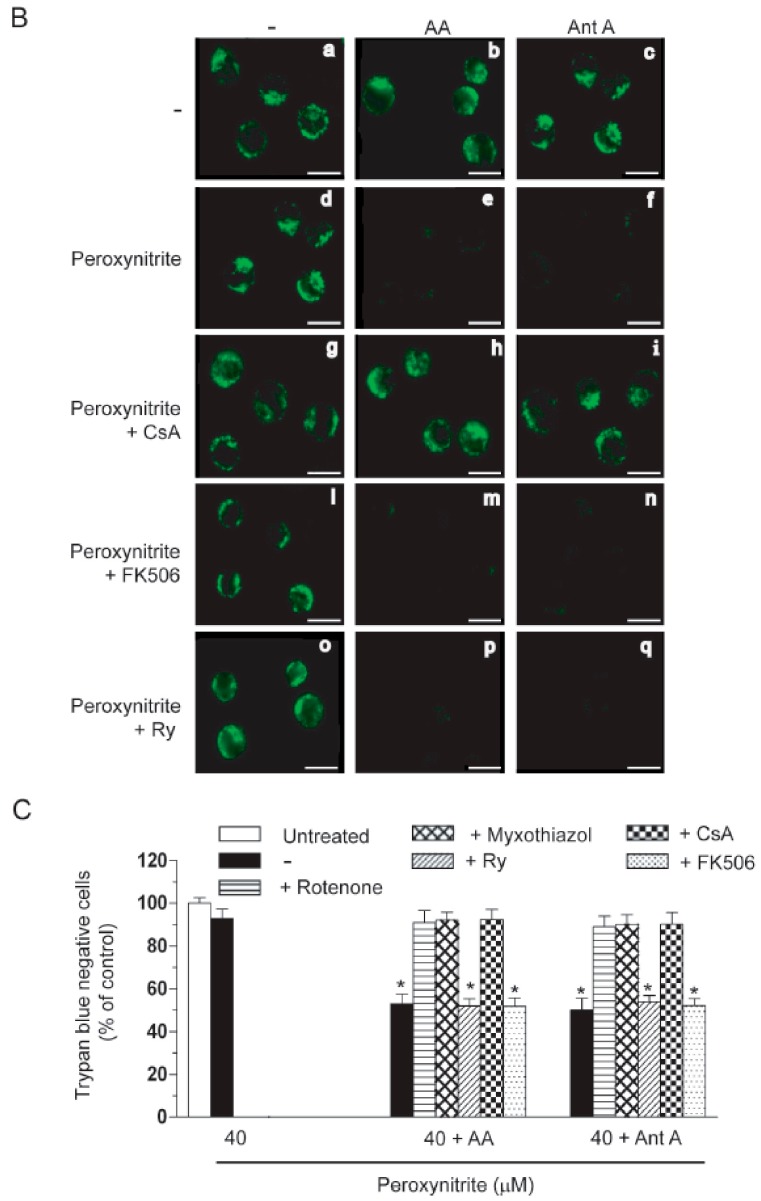
AA enhances the lethal response mediated by peroxynitrite in U937 cells via a mitochondrial permeability transition (MPT)-dependent mechanism uniquely sensitive to rotenone, myxothiazol or CsA. (**A**) U937 cells were pre-exposed for 15 min to AA, or for 5 min to antimycin A (Ant A), and then treated for 10 min with 40 μM peroxynitrite. In some experiments, rotenone, myxothiazol, Ry, CsA (0.5 µM) and FK506 (1 µM) were given to the cultures prior to peroxynitrite. After treatments, the cells were analyzed for MitoTracker red CMXRos-fluorescence; (**B**) representative micrographs of U937 cells loaded for 15 min with 1 µM calcein-acetoxymethyl ester and 1 mM CoCl_2_, washed and then post-incubated for a further 10 min with or without peroxynitrite, alone or associated with the additions indicated in the figure. The micrographs are representative of at least three separate experiments. Scale bars represent 20 µm; (**C**) cells were treated as detailed in (**A**) and then exposed for 60 min to peroxynitrite. After treatments, the cells were analyzed for toxicity with the trypan blue exclusion assay. Results represent the means ± SD calculated from at least three separate experiments. * *p* < 0.001 as compared to untreated cells (one-way ANOVA followed by Dunnett’s test).

U937 cells were exposed for 10 min to peroxynitrite and then immediately processed for the assessment of mitochondrial membrane potential (Figure 3A). While ineffective in the absence of additional treatments, the oxidant caused a significant decline in mitochondrial membrane potential (as assessed with MitoTracker red) in cells pre-exposed to AA or supplemented with antimycin A. In both circumstances, these effects were prevented by the mitochondrial permeability transition (MPT) inhibitor cyclosporin A (CsA) [21], with hardly any effect detected with FK506, which shares the ability of CsA to inhibit calcineurin, but fails to produce effects on MPT [22]. Treatments resulting in prevention of mitochondrial superoxide formation, as rotenone or myxothiazol (Figure 1A,B), also prevented the decline in mitochondrial membrane potential triggered by peroxynitrite associated with either AA or antimycin A. As a CsA-sensitive loss of mitochondrial membrane potential is indicative of the induction of MPT, we performed experiments in which MPT pore opening was assessed by monitoring the changes in mitochondrial calcein fluorescence. Cells labeled with calcein-AM display a bright and uniform fluorescence (Figure S1) that changes dramatically after addition of Co^2+^, as a result of the quenching of cytosolic and nuclear calcein-derived fluorescence. Figure 3(Ba) provides an image of untreated cells that are indeed characterized by a punctuate fluorescence, similar to the one detected with a mitochondrial probe (not shown). The results obtained with cells exposed to AA (Figure 3(Bb)), antimycin A (Figure 3(Bc)) or peroxinitrite (Figure 3(Bd)) are identical to those of untreated cells (Figure 3(Ba)), thereby providing an indication that MPT does not take place under these different treatment conditions. Exposure to peroxynitrite with either AA (Figure 3(Be)) or antimycin A (Figure 3(Bf)) instead caused a significant loss of mitochondrial fluorescence, and hence of mitochondrial calcein, indicative of mitochondrial pore opening. CsA did not produce effects in cells exposed to peroxynitrite (Figure 3(Bg)) and abolished the loss of fluorescence mediated by the oxidant in cells supplemented with either AA (Figure 3(Bh)) or antimycin A (Figure 3(Bi)). Similar results were obtained with rotenone and myxothiazol (Figure S2), whereas FK506 (Figure 3(Bm,n)) or Ry (Figure 3(Bp,q)) were ineffective under the same exposure conditions. FK506 (Figure 3(Bl)) and Ry (Figure 3(Bo)) failed to produce effects in the presence of peroxynitrite. As a final note, although not shown for the sake of brevity, it is important to state that, in Figure 3(Be,f,m,n,p,q), the presence of the cells was clearly detected by darkening the digital image at the expenses of a loss of brightness.

Collectively, the above results indicate that, otherwise inactive concentrations of peroxynitrite, induce MPT in cells supplemented with either AA or antimycin A. We finally performed experiments in which the cells were treated for 60 min and then analyzed for cytotoxicity with the trypan blue assay (Figure 3C). Peroxynitrite did not produce loss of viability, but this response was clearly detected in cells also receiving treatment with AA, or antimycin A. The mode of cell death induced by peroxynitrite/AA was remarkably similar to that previously characterized in cells exposed to the cocktail peroxynitrite/antimycin A [8]. Under both conditions, we observed the typical morphological changes of necrotic cells, eventually followed by their lysis (not shown). In addition, the lethal responses were prevented by rotenone, or myxothiazol, which also prevented mitochondrial superoxide formation (Figure 1A,B). Similar protective effects were observed after addition of CsA, under conditions associated with prevention of loss of mitochondrial membrane potential (Figure 3A) and MPT (Figure 3B). FK506, or Ry, failed to affect the lethal response mediated by peroxinitrite in association with either AA or antimycin A.

**Figure 4:**
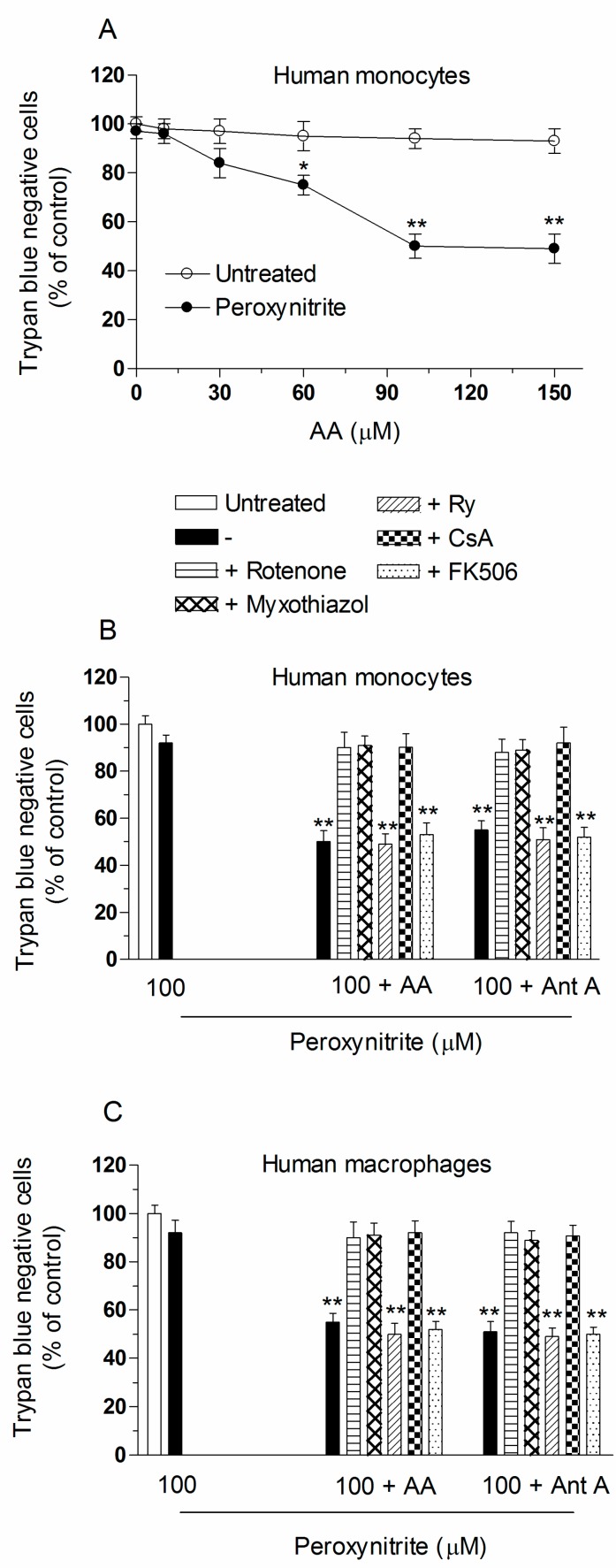
AA enhances the lethal response mediated by peroxynitrite in human monocytes or macrophages via a mechanism uniquely sensitive to rotenone, myxothiazol, or CsA. (**A**) Human monocytes were pre-exposed for 15 min to increasing concentrations of AA and then treated for a further 60 min with 0 or 100 µM peroxynitrite. After treatments, the cells were analyzed for toxicity with the trypan blue exclusion assay. Results represent the means ± SD calculated from at least three separate experiments from three different donors. * *p* < 0.01 or ** *p* < 0.001 as compared to untreated cells (two-way ANOVA followed by Bonferron’s test). Human monocytes (**B**); and macrophages (**C**) were treated as shown in the figure and then exposed for 60 min to peroxynitrite. After treatments, the cells were analyzed for toxicity with the trypan blue exclusion assay. Results represent the means ± SD calculated from at least three separate experiments using monocytes (or monocyte-derived macrophages) from three different donors. ** *p* < 0.001 as compared to untreated cells (one-way ANOVA followed by Dunnett’s test).

In other experiments using monocytes, and the treatment conditions previously shown to result in mitochondrial superoxide formation (Figure 2A) or DNA strand scission (Figure 2B), we obtained results in line with those just described for the U937 cells. We did not obtain evidence of cytotoxicity after a 60 min exposure to peroxynitrite, but a prompt lethal response was instead observed in cells supplemented with increasing concentrations of AA (Figure 4A). Once again the maximal enhancing effects were obtained using 100 µM AA. The vitamin failed to produce effects in the absence of peroxynitrite. Furthermore, the lethal response induced by the cocktail AA/peroxynitrite was identical to that mediated by antimycin A, and was in both circumstances susceptible to inhibition by rotenone, myxothiazol and CsA (Figure 4B). Cytotoxicity was instead insensitive to FK506 or Ry. Identical results were obtained using human macrophages (Figure 4C).

Collectively, the above results provide evidence for remarkable similarities in the effects mediated by AA and antimycin A, and are consistent with the notion that the vitamin enhances the toxicity mediated by peroxynitrite via Ca^2+^-independent mechanisms.

### 2.4. The Enhancing Effects of AA Are Mediated by an Increased Susceptibility of Complex III to Inhibition by Peroxynitrite

**Figure 5:**
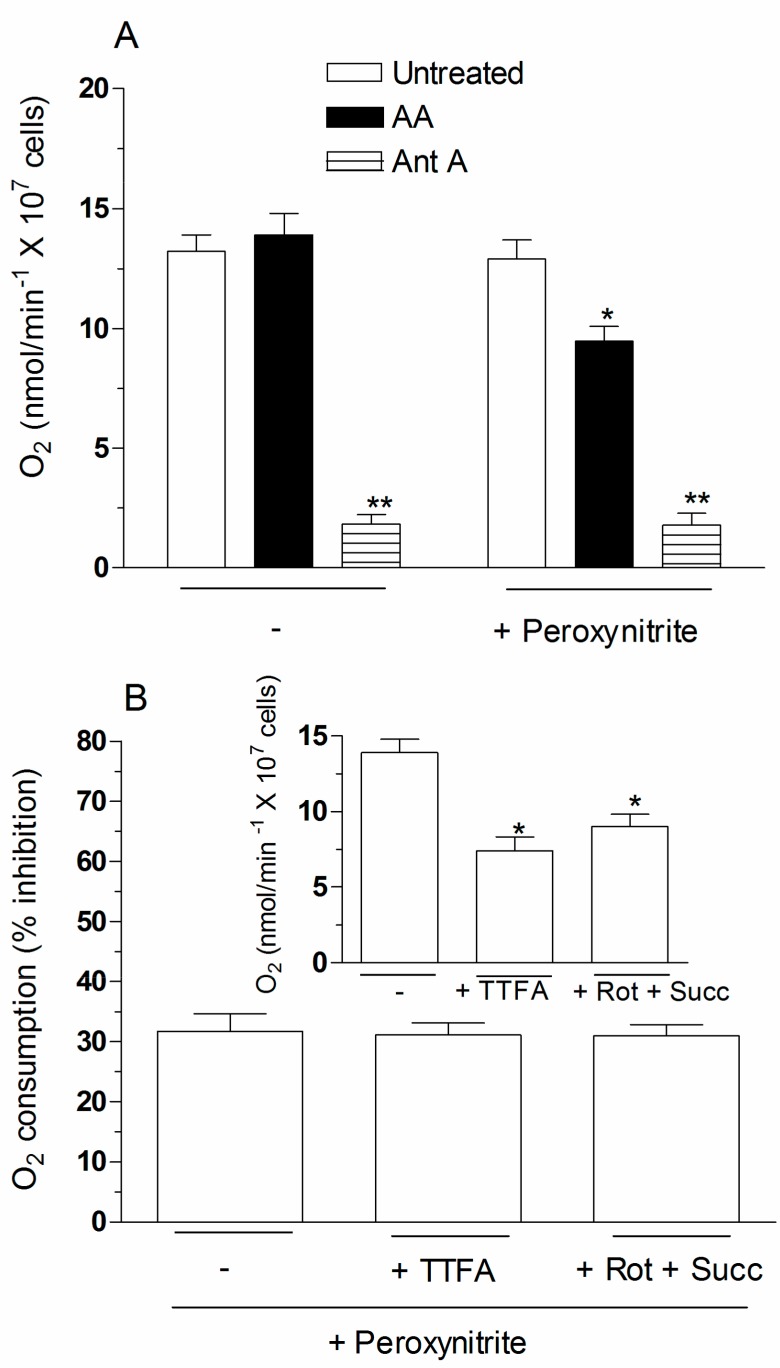

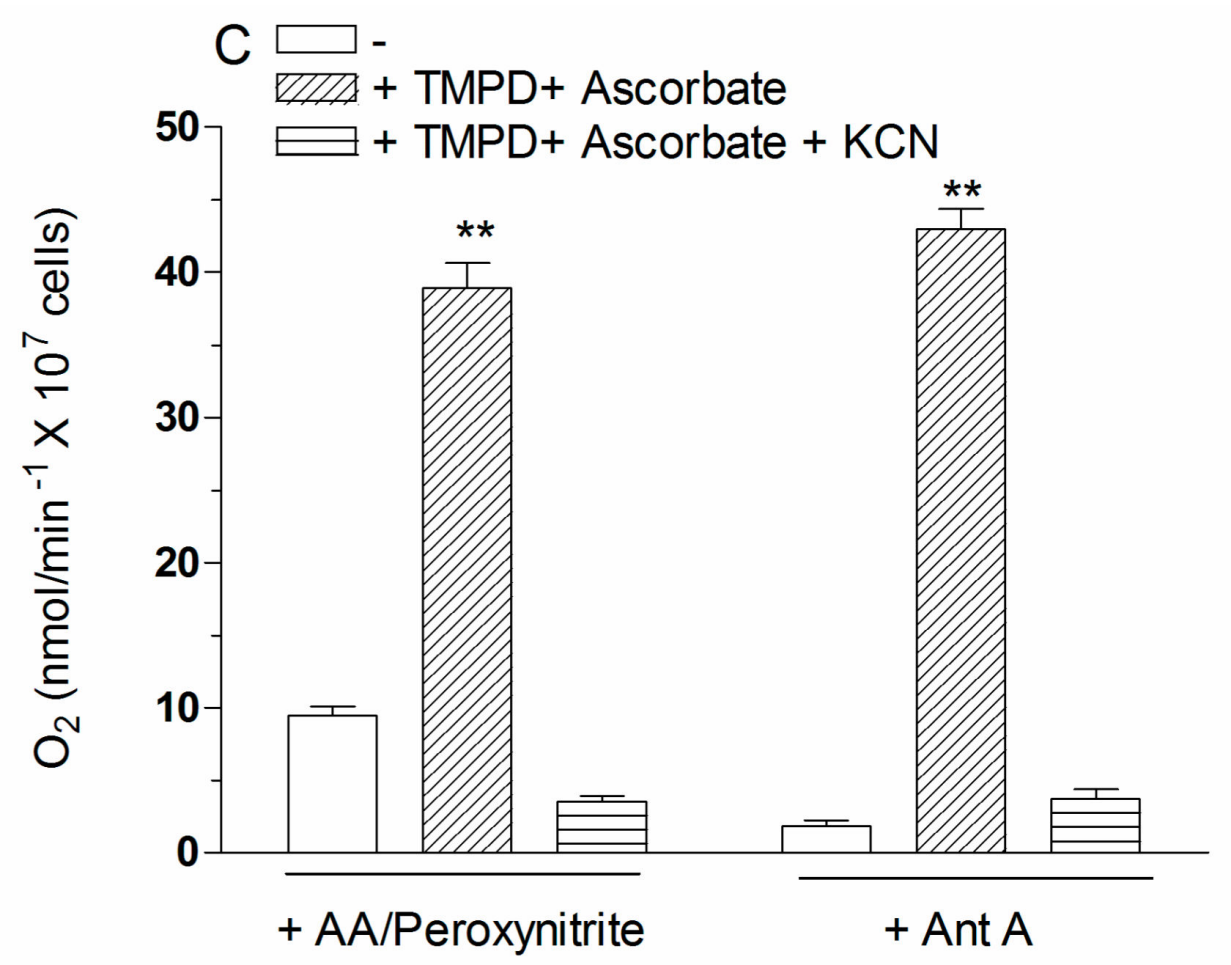
Effect of peroxynitrite on the oxygen consumption of cells preloaded with AA. (**A**) The cells were pre-exposed for 15 min to 0 or 3 µM AA and then analyzed for 3 min for oxygen consumption and for 3 more min after addition of peroxynitrite (80 µM). Oxygen consumption was also measured in cells supplemented with antimycin A (3 min) and then exposed to peroxynitrite (3 min). Results represent the means ± SD calculated from at least three separate experiments. * *p* < 0.01 or ** *p* < 0.001 as compared to untreated cells (one-way ANOVA followed by Dunnett’s test); (**B**) the cells were first pre-exposed to AA and then analysed for 3 min for oxygen consumption in the absence of other additions, or in the presence of either 250 µM TFA (trifluoroacetic acid) or 0.5 µM rotenone (Rot)/6 mM succinate (Succ) (Inset). Rotenone markedly reduced (89.13%) oxygen consumption in the absence of succinate. The extent of inhibition of oxygen consumption detected after addition of peroxynitrite (3 min) in each of these conditions was also tested (main graph). Results represent the means ± SD calculated from at least three separate experiments. * *p* < 0.01 as compared to untreated cells preloaded with AA (one-way ANOVA followed by Dunnett’s test); (**C**) the cells were pre-exposed to AA and then treated with peroxynitrite to promote inhibition of oxygen consumption, as detailed in the legend to Figure 5A. Treatment with peroxynitrite was also performed in the presence of 0.4 mM (tetramethyl-*p*-phenylenediamine) TMPD/1 mM ascorbate to directly activate complex IV. Sensitivity to KCN (1 mM) was then determined to link the increased oxygen consumption to stimulation of complex IV. For comparison, the effect of TMPD/ascorbate with or without KCN was also tested in untreated cells, in which oxygen consumption was suppressed by addition of antimycin A. Results represent the means ± SD calculated from at least three separate experiments. ** *p* < 0.001 as compared to their respective control (one-way ANOVA followed by Dunnett’s test).

The results thus far presented consistently suggest that the enhancing effects of AA are mediated by RDM, i.e., the mechanism operating in the presence of antimycin AA. As it could be easily predicted, however, the effects of the vitamin and the complex III inhibitor have a different impact on oxygen consumption. Indeed, this function was suppressed by antimycin A and hardly any effect was instead observed after addition of AA alone (Figure 5A). Based on the results obtained in this study, we should therefore postulate that AA enhances the susceptibility of complex III to the inhibitory effects mediated peroxynitrite.

To correctly address the experiments testing this possibility, we considered that cell densities used in oxygen consumption experiments are about 40 times greater than those employed in our previous experiments, and that the effects of peroxynitrite are cell density dependent [23]. Hence, the same events observed in low density cultures at a given concentration of peroxynitrite will require higher concentrations of the oxidant, in order to take place in high density cultures. We therefore made a first attempt by simply doubling the concentration of peroxynitrite. Interestingly, 80 µM peroxynitrite still failed to affect oxygen consumption, but nevertheless caused a significant (31.7%) inhibition of the same response in cells pre-exposed with AA (Figure 5A). In addition, the extent of the inhibitory response, calculated as a percentage of the respective control values (inset to Figure 5B), was comparable with that detected in complex II-inhibited cells (250 µM thenoyltrifluoroacetone (TTFA)), or cells respiring on succinate (supplemented with 0.5 µM rotenone and 6 mM succinate) (Figure 5B, main graph). Finally, similar amounts of KCN (1 mM)-sensitive oxygen consumption were observed upon addition of 0.4 mM tetramethyl-*p*-phenylenediamine (TMPD)/1 mM ascorbate to cells treated with antimycin A, or pre-exposed to AA and then treated with 80 µM peroxynitrite (Figure 5C).

These results obtained in high density cultures probably represent an underestimate of the effects of peroxynitrite, but nevertheless provide clearcut evidence for the notion that the oxidant selectively inhibits complex III in AA-preloaded cells, under conditions in which complex I, II or IV remain unaffected. Hence, mitochondrial AA promotes events mediated by peroxynitrite at the complex III level, thereby causing inhibition of oxygen consumption and parallel superoxide formation.

## 3. Discussion

The U937 cell clone employed in our laboratory can be conveniently used in studies investigating the intramitochondrial effects of AA since these cells, as summarized in the Introduction Section, avidly take up the vitamin in the cytosol and then in their mitochondria [13,18]. This second process appears particularly efficient, since mediated by a transporter recognized by anti-SVCT2 antibodies working with a high affinity even at low Na^+^ concentrations and in the virtual absence of Ca^2+^ [13].

We recently used these cells to demonstrate that the mitochondrial fraction of AA is responsible for the prevention of mitochondrial superoxide formation elicited by arsenite [24]. This effect is consistent with the well-established notion that AA is a reducing agent, an antioxidant and a scavenger of various reactive species [9,10]. However, besides inducing the above protective effects, the vitamin can interact with redox active metal ions (normally found in most culture media [25,26]) and hence behave as a pro-oxidant [25,27]. These conditions generally require high concentrations of the vitamin and the resulting cytotoxicity is mediated by the formation of superoxide and hydrogen peroxide. Intravenous administration of pharmacological concentrations of vitamin C also promotes cancer cell death in experimental animals [28] and in humans [29]. In addition, AA can be engaged in specific reactions leading to enhanced responses to specific reactive species [11,12,30,31,32]. As an example, we found that pre-exposure of U937 cells to AA enhances their susceptibility to the deleterious effects mediated by various hydroperoxides, in particular peroxynitrite [11,12]. This reactive nitrogen species, at intrinsically active concentrations, promotes the mitochondrial formation of superoxide via CRDM, since requiring Ca^2+^ as well as inhibition of electron transport. Sub-optimal levels of peroxynitrite may also promote maximal mitochondrial superoxide formation through either CDM, uniquely dependent on the mitochondrial Ca^2+^ accumulation triggered by RyR agonists [5,6], or RDM, taking place under conditions associated with suppression of electron transport, secondary to inhibition of complex III [4,6]. The enhancing effects mediated by AA in cells exposed to peroxynitrite and other oxidants were also described in other laboratories [30,31,32]. Although it is difficult to extrapolate the significance of experimental results obtained with cultured cells to draw conclusions relevant to human physio-pathology, reports in the literature describe deleterious effects mediated by acute supplementation of AA to individuals affected by β-thalassaemia major [33], as well as idiopathic haemochromatosis and dietary iron overload [34,35]. Similar responses associated to exposure of vitamin C were documented in an acute human inflammatory model associated with eccentric exercise [36,37] and in sepsis [38].

The present study was performed with the aim of establishing the mechanism involved in the enhancing effects mediated by AA in cells exposed to otherwise inactive concentrations of peroxynitrite. Several lines of evidence consistently indicated that the enhanced mitochondrial formation of superoxide, triggering various downstream events as DNA damage, or mitochondrial dysfunction, is Ca^2+^-independent and in fact uniquely mediated by a mechanism resulting in inhibition of electron transport in the respiratory chain. This conclusion is supported by the following observations:

- The enhancing effects of AA, detected in terms of mitochondrial superoxide formation and downstream DNA strand scission, were sensitive to rotenone, or myxothiazol, as in the case of CRDM and RDM, but not CDM.
- The enhancing effects of AA were insensitive to Ry, as for RDM, but not CRDM or CDM.
- There was no evidence of increased mitochondrial accumulation of Ca^2+^ after treatment with AA and/or peroxynitrite, as in the case of RDM. Increased mitochondrial accumulation of Ca^2+^ was instead associated with either CRDM or CDM.
- The DNA strand scission mediated by peroxynitrite in cells permeabilized after pre-exposure to AA, while suppressed by rotenone or myxothiazol, was insensitive to Ry, EGTA or inhibitors of mitochondrial Ca^2+^ uptake. All these treatments were instead effective in CRDM.
- Mitochondrial superoxide formation and DNA strand scission induced by peroxynitrite were also enhanced by AA in human monocytes and macrophages, resistant to both CRDM and CDM since not expressing the RyR [6,7].

These results indicate that intramitochondrial AA promotes the peroxynitrite-dependent mitochondrial formation of superoxide via a Ca^2+^-independent mechanism, identical to that previously described for antimycin A. The same notion was established by measuring another endpoint, DNA strand scission, implying the conversion of superoxide to H_2_O_2_ and the migration of the latter to the nucleus. Importantly, however, the effects of the vitamin were not associated with direct inhibition of oxygen consumption, as it occurs in cells supplemented with antimycin A [4]. In fact, the mitochondrial fraction of AA enhanced the sensitivity of complex III of the respiratory chain to the low dose of peroxynitrite. Although more studies are necessary to understand the molecular bases of these enhancing effects, one likely possibility is that intramitochondrial AA promotes conversion of trivalent iron to its redox active divalent form in critical sites of complex III. Divalent iron is then involved in the site-specific generation of reactive species, enforcing events associated with further superoxide formation. Clearly, an important determinant of this response is represented by the high intramitochondrial concentration of the vitamin, which in U937 cells is supported by a high expression of mitochondrial SVCT2. We can easily predict that a less efficient mitochondrial transport of the vitamin will reduce the susceptibility of the cells to the Ca^2+^-independent mechanism leading to superoxide formation. A good example is provided by our experiments using human monocytes and macrophages, showing that one order of magnitude greater concentrations of AA are required to promote the same enhancing effects detected in U937 cells. A final important observation was that the loss of mitochondrial function and integrity, and the ensuing lethal response, detected in U937 cells as well as human monocytes and macrophages supplemented with AA and peroxynitrite, were also Ca^2+^-independent. This is an interesting observation since the Ca^2+^-dependence of MPT has been univocally established in most toxicity paradigms [39,40].

**Figure 6:**
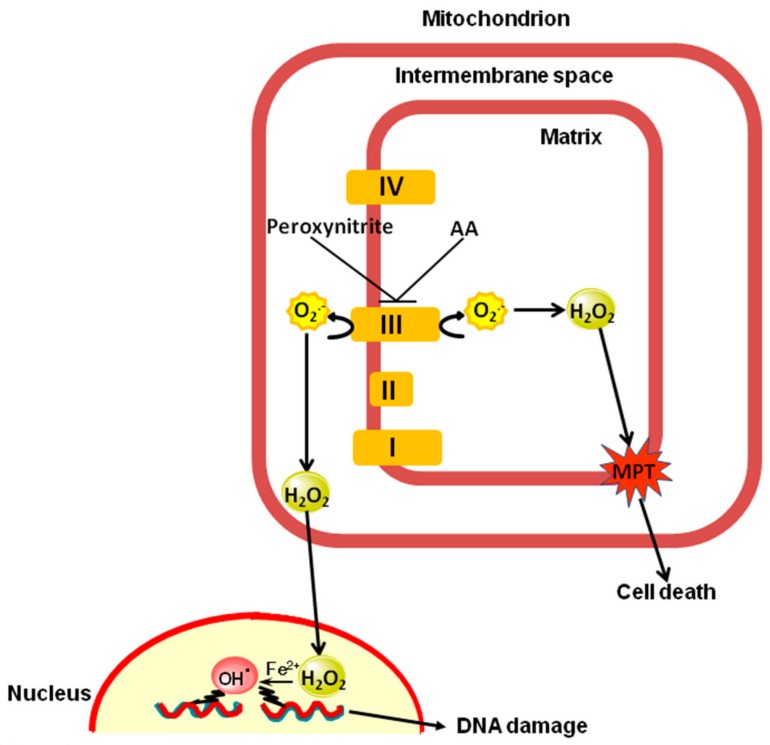
The enhancing effects mediated by the mitochondrial fraction of AA in cells exposed to peroxynitrite: proposed mechanism. AA increases the vulnerability of complex III to peroxynitrite via a Ca^2+^-independent mechanism. The extramitochondrial effects observed under these conditions are caused by superoxide released by complex III in the intermembrane space, which readily dismutates to a diffusible species, H_2_O_2_. The intramitochondrial effects are instead mediated by superoxide formation in the matrix followed by its conversion to H_2_O_2_.

Our previous studies underscoring the existence of CRDM [4,6], CDM [5,6] and RDM [4,6,8] are therefore now implemented by the demonstration that this last mechanism is not only triggered by bona fide complex III inhibitors and is not always associated with suppressed oxygen consumption. This observation furthers our knowledge on the complexity of the mechanisms whereby peroxynitrite, or other lipid hyperoxides, indirectly mediate damage in target molecules. More specifically, we demonstrate the mitochondrial fraction of vitamin C enhances the effects of peroxynitrite on complex III via a Ca^2+^-independent mechanism to promote mitochondrial superoxide formation, and downstream strand scission of genomic DNA, as well as mitochondrial dysfunction leading to MPT-dependent cytotoxicity. Figure 6 summarizes these findings and provides an integrated mechanism based on the notion that complex III releases O_2_^−^ in the intermembrane space as well as in the matrix [41].

## 4. Materials and Methods

### 4.1. Chemicals

AA, antimycin A, Cf, rotenone, myxothiazol, Ry, RR, LaCl_3_, EGTA, TTFA, TMPD, KCN and the remaining chemicals were from Sigma-Aldrich (Milan, Italy). CsA was from Novartis (Bern, Switzerland). FK506 was obtained from Calbiochem (San Diego, CA, USA). MitoSOX red, Rhod 2-AM, MitoTracker Red CMXRos and calcein AM were from Molecular Probes (Leiden, The Netherlands).

### 4.2. Cell Culture and Treatments

U937 human myeloid leukemia cells were cultured in suspension in RPMI 1640 medium (Sigma-Aldrich) supplemented with 10% fetal bovine serum (Euroclone, Celbio Biotecnologie, Milan, Italy), penicillin (100 units/mL) and streptomycin (100 µg/mL) (Euroclone), at 37 °C in T-75 tissue culture flasks (Corning, Corning, NY, USA) gassed with an atmosphere of 95% air-5% CO_2_.

Human blood samples in heparinized vacutainers were obtained from healthy volunteers included in the list of blood donors from the Blood Transfusion Center of the Hospital “S. Maria della Misericordia”, Urbino (PU) Italy after signing a written informed consent, under a protocol approved by the Ethical Committee of the University of Urbino “Carlo Bo” (Protocol Number: 6589, approved on 16 June 2010).

Human peripheral mononuclear cells were isolated by Ficoll gradient centrifugation, and monocytes were purified by adherence in RPMI-medium. Non-adherent cells were removed by repeated washing with phosphate-buffered saline (136 mM NaCl, 10 mM Na_2_HPO_4_, 1.5 mM KH_2_PO_4_, 3 mM KCl; pH 7.4) while adherent cells were subsequently scraped with trypsin, and then utilized for monocyte experiments. Macrophages were obtained by culturing monocytes (1 × 10^6^ cells/mL) for 8–10 days in RPMI-medium. Macrophages were scraped with trypsin, centrifuged and utilized for experiments.

A 10 mM AA stock solution was prepared in extracellular buffer (15 mM Hepes, 135 mM NaCl, 5 mM KCl, 1.8 mM CaCl_2_, 0.8 mM MgCl_2_, pH 7.4) containing 100 µM DTT, immediately before utilization. Cells were re-suspended in extracellular buffer (1 × 10^6^ cells/mL) and exposed to AA. Under these conditions, AA remained stable, as assessed by monitoring (for at least 15 min) the absorbance at 267 nm (ε_267_ = 14,600 M^−1^ cm^−1^).

Stock solutions of Cf, RR, LaCl_3_ and catalase were freshly prepared in distilled water. Antimycin A, Ry and myxothiazol were dissolved in 95% (*v*/*v*) ethanol. Rotenone was dissolved in dimethyl sulfoxide. At the treatment stage the final ethanol or dimethyl sulfoxide concentrations were never higher than 0.05%. Under these conditions ethanol, or dimethyl sulfoxide, was neither toxic nor DNA-damaging, nor did it affect the cyto-genotoxic properties of peroxynitrite.

Experiments with intact cells were performed using 15 mL plastic tubes containing 2.5 × 10^5^ cells/mL of pre-warmed saline A (140 mM NaCl, 5 mM KCl, 4 mM NaHCO_3_, and 5 mM glucose; pH 7.4). Similar conditions were used in experiments employing permeabilized cells. Permeabilization was achieved by adding digitonin (10 µM) to a medium consisting of 0.25 M sucrose, 0.1% (*w*/*v*) bovine serum albumin, 10 mM MgCl_2_, 10 mM K^+^-Hepes, 5 mM KH_2_PO_4_, pH 7.2 at 37 °C. Under these conditions, digitonin permeabilizes the plasma membrane but leaves mitochondrial membranes intact [42].

Peroxynitrite, synthesized as previously described [23], was rapidly added on the wall of the plastic tubes and mixed to equilibrate the peroxynitrite concentration on the culture medium. To avoid changes in pH due to the high alkalinity of the peroxynitrite stock solution, an appropriate amount of 1.5 N HCl was also added to the wall of the tubes prior to peroxynitrite.

### 4.3. Measurement of AA Content

After treatments, the cells were washed twice with ice-cold extracellular buffer and the final pellets were extracted with ice-cold 70% (*v*/*v*) methanol/30% solution (10 mM tetrabutylammonium hydrogen sulfate, 10 mM KH_2_PO_4_, 0.5% methanol, pH 6.0) containing 1 mM ethylenediaminetetraacetic acid and 10 mM DTT. After 10 min at ice bath temperature, the samples were centrifuged at 10,000× *g* for 20 min at 4 °C. Samples were filtered through a 0.22 μm filter (Millipore Corporation, Billerica, MA, USA) and either analyzed immediately or frozen at −80 °C for later analysis. AA content was measured by HPLC with the UV detection wavelength set at 265 nm, as described in [43], with minor modifications. The assay involved the use of a 15 cm × 4.6 mm Discovery C-18, 5 μm column (Supelco, Bellefonte, PA, USA) equipped with a Supelguard Discovery C-18 guard column (2 cm × 4 mm, 5 μm). The injection volume was 20 μL. Under these conditions the retention time of AA was about 4 min. AA concentration was determined from the corresponding calibration curve constructed with the pure chemical AA dissolved in the extraction solution.

The same procedure was performed to evaluate AA content in mitochondria isolated from cells previously exposed to AA. The mitochondrial fraction was isolated as detailed in [44].

Intracellular and intramitochondrial concentrations of AA were calculated using published values for cell [45] and mitochondrial [46] volumes, respectively.

### 4.4. MitoSOX Red Oxidation

Cells were first pre-loaded with AA and subsequently exposed for 15 min (37 °C) to 5 µM MitoSOX red (Molecular Probes) in 35 mm tissue culture dishes containing an uncoated coverslip. Under these conditions, U937 cells attach to the glass substrate. Dishes were then washed twice with saline A and cells treated with peroxynitrite, as detailed in the legend to the figures. Coverslips were finally washed three times and fluorescence images were captured with a BX-51 microscope (Olympus, Milan, Italy), equipped with a SPOT-RT camera unit (Diagnostic Instruments, Delta Sistemi, Rome, Italy) using an Olympus LCAch 40×/0.55 objective lens (Olympus). The excitation and emission wavelengths were 510 and 580 nm with a 5-nm slit width for both emission and excitation. Images were collected with exposure times of 100–400 ms, digitally acquired and processed for fluorescence determination at the single cell level on a personal computer using Scion Image software (Scion Corp., Frederick, MD, USA). Mean fluorescence values were determined by averaging the fluorescence values of at least 50 cells/treatment condition/experiment.

### 4.5. Aconitase Activity

After treatments with peroxynitrite (10 min), the cells were washed twice with saline A, resuspended in lysis buffer (50 mM Tris-HCl, 2 mM Na-citrate, 0.6 mM MnCl_2_, pH 7.4) and finally sonicated three times on ice by using the Sonicator Ultrasonic Liquid Processor XL (Heat System-Ultrasonics, Inc., Jamestown, NY, USA) operating at 20 W (30 s). The resulting homogenates were centrifuged for 5 min at 18,000× *g* at 4 °C. Aconitase activity was determined spectrophotometrically in the supernatants at 340 nm, as described by Gardner [17].

### 4.6. Measurement of DNA Single-Strand Breakage by the Alkaline Halo Assay

DNA single-strand breakage was determined immediately after exposure to peroxynitrite (30 min) with the alkaline halo assay [47]. It is important to keep in mind that, although we refer to DNA strand scission throughout the text, the DNA nicks measured by this technique under alkaline conditions may in fact include alkali labile sites in addition to direct strand breaks. Details on the alkaline halo assay and processing of fluorescence images and on the calculation of the experimental results are also given in [47]. DNA single-strand breakage was quantified by calculating the nuclear spreading factor value, representing the ratio between the area of the halo (obtained by subtracting the area of the nucleus from the total area, nucleus + halo) and that of the nucleus, from 50 to 75 randomly selected cells/experiment/treatment condition. Results are expressed as relative nuclear spreading factor values calculated by subtracting the nuclear spreading factor values of control cells from those of treated cells.

### 4.7. Cytotoxicity Assay

After treatments with peroxynitrite (60 min), the number of viable cells was estimated with the trypan blue exclusion assay. Briefly, an aliquot of the cell suspension was diluted 1:2 (*v*/*v*) with 0.4% trypan blue and the viable cells (i.e., those excluding trypan blue) were counted with a hemocytometer.

### 4.8. Measurement of Mitochondrial Ca^2+^

Cells were first exposed for 30 min (4 °C) to 10 µM Rhod 2-acetoxymethyl ester, washed three times with saline A and finally incubated for 5 h in RPMI 1640 medium (37 °C). This two-step cold loading/warm incubation protocol achieves loading of Rhod 2 into the mitochondria [48]. Cells were pre-loaded with vitamin C and subsequently manipulated to attach on a coverslip, as indicated for experiments measuring MitoSOX red fluorescence. Fluorescence images were visualized using a fluorescence microscope and the resulting images were taken and processed as described above. The excitation and emission wavelengths were 540 and 590 nm, respectively, with a 55-nm slit width for both emission and excitation. Mean fluorescence values were determined by averaging the fluorescence values of at least 50 cells/treatment condition/experiment.

### 4.9. MitoTracker Red CMXRos, Calcein Staining and Imaging

Cells were pre-loaded with AA and treated for 3 min (37 °C) in saline A with peroxynitrite in 35 mm tissue culture dishes containing an uncoated coverslip. Subsequently, the cells were post-incubated for a further 7 min with various additions and 50 nM MitoTracker Red CMXRos.

In other experiments the cells were incubated for 15 min (37 °C) in saline A with 1 μM calcein-acetoxymethyl ester and 1 mM CoCl_2_, washed twice with saline A and treated with peroxynitrite (10 min), as detailed in the legend to the figures. After treatments, the cells were washed three times and analyzed with a fluorescence microscope. The resulting images were taken and processed as described above. The excitation and emission wavelengths were 488 and 515 nm (calcein) and 545 and 610 nm (MitoTracker Red CMXRos), with a 5-nm slit width for both emission and excitation. Images were collected with exposure times of 100–400 ms, digitally acquired and processed for fluorescence determination at the single cell level on a personal computer using the J-Image software. Mean fluorescence values were determined by averaging the fluorescence values of at least 50 cells/treatment condition/experiment.

### 4.10. Measurement of Oxygen Consumption

The cells were washed once in saline A and then re-suspended in the same medium at a density of 1 × 10^7^ cells/mL. Oxygen consumption was measured using a YSI oxygraph equipped with a Clark electrode (model 5300, Yellow Springs Instruments, Yellow Springs, OH, USA). The cell suspension (3 mL) was transferred to the polarographic cell and the rate oxygen utilization was monitored under constant stirring for 3 min (basal respiration). The rate of oxygen utilization was calculated as described previously [49].

### 4.11. Statistical Analysis

The results are expressed as means ± SD. Statistical differences were analyzed by one-way ANOVA followed by Dunnett’s test for multiple comparison or two-way ANOVA followed by Bonferroni’s test for multiple comparison. A value of *p* < 0.05 was considered significant.

## 5. Conclusions

Our results demonstrate that the mitochondrial fraction of vitamin C enhances the susceptibility of U937 cells to the deleterious effects mediated by peroxynitrite via a Ca^2+^-independent mechanism. Under these conditions, otherwise inactive concentrations of the oxidant caused extensive mitochondrial superoxide formation, and downstream DNA strand scission, as well as mitochondrial dysfunction leading to MPT-dependent cytotoxicity. The enhancing effects of AA are therefore mediated by a mechanism that can be classified as RDM, as previously reported with the complex III inhibitor antimycin A [4,6,8]. However, AA did not directly inhibit complex III but rather enhanced its susceptibility to the inhibitory effects of the low dose of peroxynitrite. As a final note, the notion of the Ca^2+^-independence of the effects associated with superoxide formation should also be extended to the MPT elicited by the cocktail AA/peroxynitrite.

## Supplementary Materials

Supplementary materials can be found at www.mdpi.com/1422-0067/18/8/1686/s1.
