# Supplementary material for: Intramitochondrial Ascorbic Acid Enhances the Formation of Mitochondrial Superoxide Induced by Peroxynitrite via a Ca2+-Independent Mechanism
Source: Int J Mol Sci. 2017 Aug 2;18(8):1686. doi: 10.3390/ijms18081686 (PMC5578076; doi:10.3390/ijms18081686)
Supplement: Supplementary file 1 [file ijms-18-01686-s001.pdf]

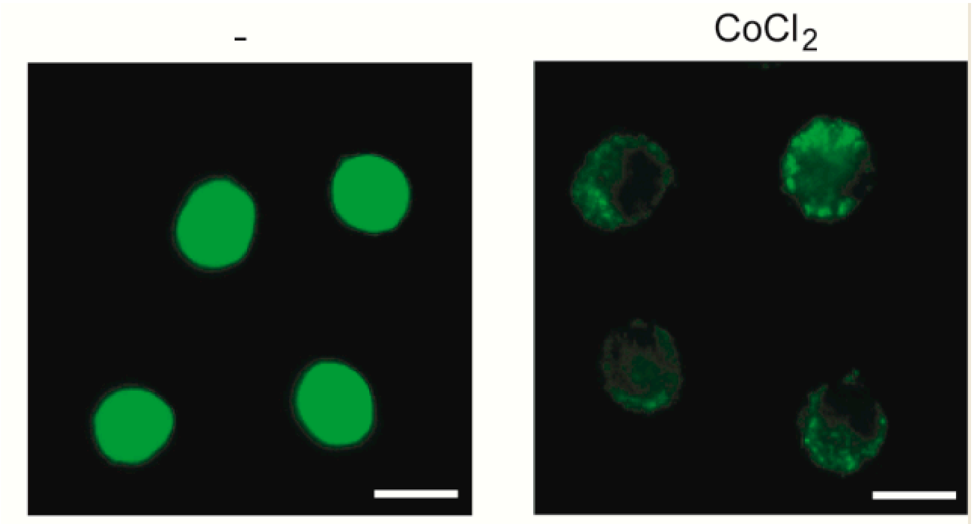

Figure S1. Representative micrographs of U937 cells loaded for 15 min with 1 μM calcein-acetoxymethyl ester alone, or associated with 1 mM $CoCl_2$, washed and then post-incubated for a further 15 min in fresh saline A. The micrographs are representative of at least three separate experiments with similar outcomes. Scale bars represent 20 μm.

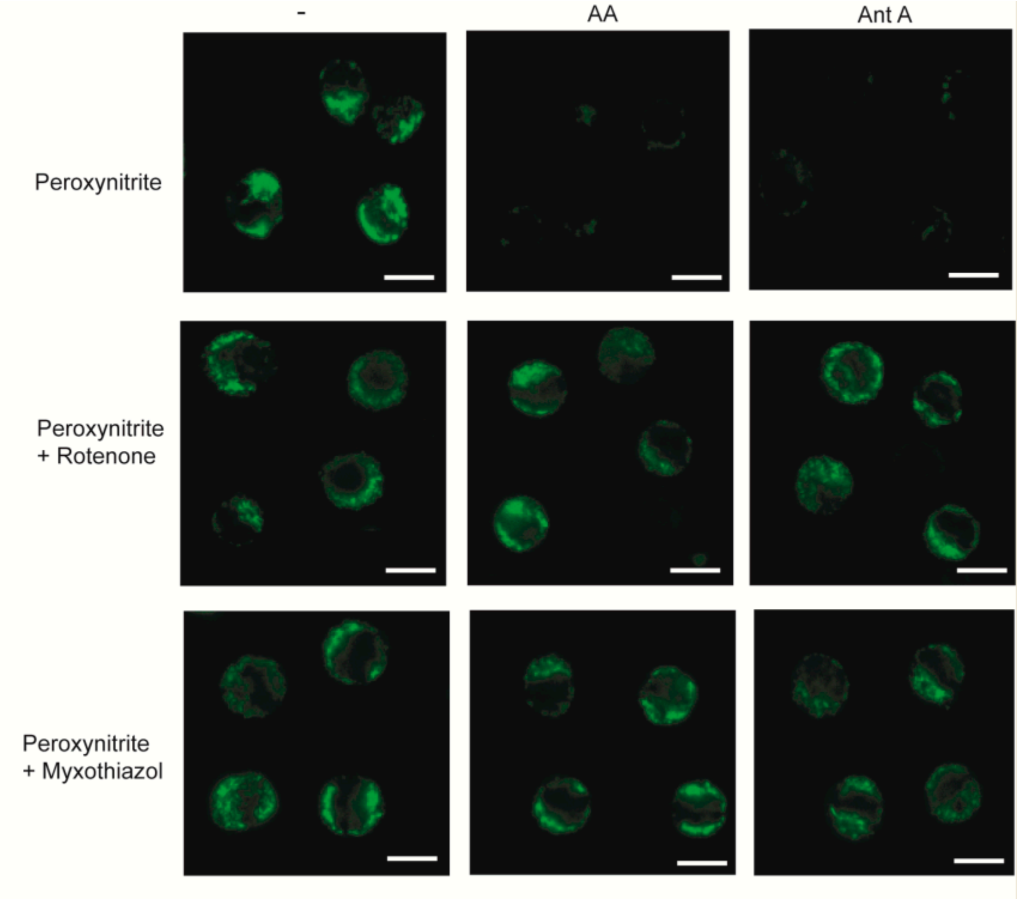

Figure S2. Rotenone, or myxothiazol, prevents mitochondrial permeability transition (MPT) induced by peroxynitrite in AA- or antimycin A-supplemented cells. Representative micrographs of U937 cells loaded for 15 min with 1 μM calcein-acetoxymethyl ester and 1 mM $CoCl_2$, washed and then post-incubated for a further 10 min with or without peroxynitrite, alone or associated with the additions indicated in the figure. The micrographs are representative of at least three separate experiments. Scale bars represent 20 μm.
